# Effects of Robot-Assisted Therapy on Gait Parameters in Pediatric Patients With Spastic Cerebral Palsy

**DOI:** 10.3389/fneur.2021.724009

**Published:** 2021-12-23

**Authors:** Faustyna Manikowska, Sabina Brazevic, Anna Krzyżańska, Marek Jóźwiak

**Affiliations:** ^1^Gait and Motion Analysis Laboratory, Poznań University of Medical Sciences, Poznań, Poland; ^2^X-Rehab, Poznań Rehabilitation Center, Poznań, Poland

**Keywords:** gait analysis, balance, symmetry index, gait deviation index, spatiotemporal parameters

## Abstract

**Background:** Gait dysfunction is a crucial factor that restricts independence and quality of life in children with cerebral palsy (CP). Gait training based on robotic-assisted therapy (RAT) is widely used, but information about effectiveness and ideal patient profile is not sufficient. Aim of this study was to assess the effect of RAT on gait parameters in spastic children with CP, and to determine whether changes in gait parameters are different among patients on different ambulatory levels.

**Method:** A total of 26 children with bilateral spastic CP were divided into two groups based on their functional ability: non-assisted ambulator (NAS) or assisted ambulator (AS); and underwent a RAT program (30 training sessions of RAT during 10 weeks). Gait analysis was performed: before the therapy (t1), right after (t2), and 6 weeks later (t3).

**Results:** No significant changes in spatiotemporal parameters or gait deviation index at t2 or t3. Double support symmetry significantly improved (t1 vs. t3, *p* = 0.03) for the whole group (NAS + AS). Walking speed symmetry significantly improved (t2 vs. t3, *p* = 0.02) for group AS.

**Conclusion:** RAT based on our protocol did not change spatiotemporal parameters and kinematics of walking except limited improvement in some aspects of gait symmetry. We did not find differences in changes in selected objective gait parameters among children with CP in different ambulatory levels.

## Introduction

Cerebral palsy (CP) is a disorder with a non-homogeneous combination of symptoms that leads to disability. In most cases, the mixture of positive (increased muscle activities) and negative (insufficient active muscle control) signs of motor disorders can be presented (1, 2). Various combinations of simultaneous present and severity of impairments are observed as the clinical conditions of an individual subject and make an impact on their functional status (3). Because of that, therapy for this population should be individualized and task-oriented for each patient (4).

Gait dysfunction is a crucial factor that restricts independence and quality of life (5–7). Meta-analysis has shown that gait training is the most effective intervention to improve gait parameters in patients with CP (8). The robot-assisted therapy (RAT) has been combined to the conventional gait training recently (9). It has been developed and improved to meet the needs of each patient. Based on the real-time feedback and collected data, individual differences on therapy, including time, endurance, and level of advance can be adapted (10, 11). It was suggested that robot-assisted gait training is an effective therapy for this population (8, 12–14).

Despite the fact that the new generation of RAT can meet individual requirements better than conventional training in theory and may lead to better functional outcomes, the amount and quality of research in this field is still very limited (8). Training protocols mainly focused on gait training and outcomes measures of the therapy differ from study to study (12–15). Most of the studies focused on walking endurance, walking speed, and functional assessment of walking ability with varying numbers of therapy sessions from 1 to 20 (16, 17). Moreover, small sample size is a very common limitation (14, 18–20).

The primary aim of the study was to assess the effect of RAT on selective objective gait parameters in children with spastic CP. The secondary aim was to determine whether changes in selected objective gait parameters are different among children in different ambulatory levels. To achieve the aforementioned aims, we investigated the short-term changes in gait kinematic data, gait symmetry, and gait spatiotemporal parameters from gait analyses performed before and after RAT, in the two functional groups: independent ambulators and dependent ambulators who use assistive devices.

## Materials and Methods

### Participants

Children were recruited to participate in this prospective study. Inclusion criteria were: (1) diagnosis of bilateral spastic CP, (2) able to follow verbal instructions, (3) gait training was the aim of the therapy, (4) body height at least 150 cm, and (5) only received conventional therapy within the past 6 months. Exclusion criteria were: (1) received surgery or botulinum toxin injection within the past 6 months, (2) pain or fixed contractures in the lower limb joints that prevent from applying RAT.

In total, 26 children with bilateral spastic CP were recruited to the study (female, *n* = 10; male, *n* = 16; age = 14.8 [1.97] years; range: 12–18 years). All the participants underwent a RAT program in the outpatient service of a local rehabilitation hospital and were examined in the motion analysis laboratory of the university hospital. Participants were divided into two groups based on their baseline functional ability, that is, Gross Motor Function Classification System (GMFCS) level, before the therapy program started: Group NAS (non-assisted ambulator, *n* = 17) were those who could walk independently (GMFCS levels I and II), and Group AS (assisted ambulator, *n* = 9) were those who walk with assistive devices (GMFCS levels III & IV).

The study received appropriate approval from the Institutional Review Board of the Poznan University of Medical Sciences (nr 681/18, June 16, 2018). Written consents were acquired from all the participants. For those younger than 18 years, consents were obtained from the legal guardian.

### Protocol

Anthropometric data (age, body weight, and body height) were collected from all the participants at their first visit.

#### Robot-Assisted Therapy Program (Gait Training) Protocol

Each patient received a 10-week (30 sessions) RAT protocol in the following order: training (2 weeks; five sessions per week), break (2 weeks), training (2 weeks; five sessions per week), break (2 weeks), training (2 weeks; five sessions per week). This therapy protocol was created based on the Camp's formula in order to assess the effectiveness of an intensive, complex, and long-term RAT, and to monitor physical activities and the physical therapy during the time the present study was conducted. The 2-week break allows participants to return their residential place temporarily because of emotional, family, and employment needs.

During the first session, the participants got familiar with the therapy program. On the 1st day, the therapy dose was optimized to meet individual capability of each patient. At each equipment, the diagnostic tests were done. Balance, ability to shift the center of pressure, spatiotemporal gait parameters, endurance, and the level of required support during walking were assessed. Based on these data, the baseline level for each patient was established and it was a starting point for individual therapy. During the RAT based on the real-time assessment (based on the same diagnostic tests) the progress was detected and the training difficulty was adjusted. Virtual reality games were integrated into treadmill and platforms. The amount of support for the exoskeleton system was established on the first session. Based on the feedback information from each lower limb joint, the support level for each leg was established and adjusted.

The functional gait training was based on RAT. Because the dynamic balance influences walking pattern, we decided that each session consists of: gait training (with exoskeleton and treadmill) and balance training (on stabilometric and dynamographic platforms) according to the therapy protocol:

- 10 min on Gamma VAST (AC International East):
  ■ analysis of load distribution between left and right sides of the body side (for balance training)
  ■ create individual training difficulty by the real-time biofeedback
- 5-min break
- 10 min on Alfa VAST (AC International East):
  ■ dynamic analysis of center of pressure displacement
  ■ training of balance according to amount of displacement
- 10-min break
- 45 min of EksoGT (Ekso Bionics):
  ■ analysis of required support of each lower limb joint
  ■ customized gait training with different level of support.

The training starts from a shorter period of time (10–15 min). Depends on the endurance, the usual walking time range from 30 min to 1 h.

- 15-min break
- 2 × 15 min on zebris THQ-M-3i Treadmill (zebris Medical GmbH)
  ■ analysis of spatiotemporal gait parameters and endurance
  ■ virtual reality trainings for walking balance and gait
- 5-min break

The whole therapy program was performed under the supervision of a physical therapist experienced with RAT.

#### Gait Analysis

Gait analysis was performed at the following three time points: before the therapy program started (t1), right after all 30 training sessions were completed (t2), and 6 weeks after the therapy program was done (t3).

Kinematic data were collected with an eight-camera three-dimensional gait analysis system (six Bonita cameras and two Vero cameras; Vicon Motion Systems Ltd., Oxford, United Kingdom) sampling at 100 Hz. Reflective markers were applied according to the standard Plug-in-Gait marker placement model to each patient. Participants walk barefoot along a 10-m walkway with self-selected speed.

#### Data Analysis

##### Statistical Analysis

The normality of the distribution of variables was tested with the Shapiro-Wilk test. To investigate the changes over time of the analyzed parameters, in case of compliance with the normal distribution and the homogeneity of variance, repeated-measures ANOVA with Tukey's multiple comparisons test was calculated. In the remaining cases, the Friedman test with the Dunn-Bonferroni multiple comparison test was calculated. In order to assess whether the changes in the parameters analyzed had deterioration, no change, improvement over time are statistically significant, the McNemar-Bowker symmetry test was used. To compare the data between ambulators and non-ambulators, *t*-test, Cochran-Cox test, or Mann-Whitney test was used depending upon the distribution of normality and variance equality. A power analysis was performed to estimate the appropriate sample size. All the calculations were made using Statistical version 12 (TIBCO Software Inc., Palo Alto, California, USA). Statistical significance was set at the *p*-value of 0.05.

##### Outcome Measure

Primary outcome measures were: spatiotemporal gait parameters, including walking speed (m/s), cadence (steps/min), step time (s), step length (m), stride time (s), stride length (m), step width (m), foot off (% of the gait cycle), double support (% of the gait cycle), single support (% of the gait cycle), opposite foot off (% of the gait cycle), and opposite foot contact (% of the gait cycle). All spatiotemporal parameters were processed in the default data collection software provided by the instrumental gait analysis system manufacturer (Vicon Motion Systems Ltd., Oxford, UK) and extracted directly from the standard C3D format file from a custom-coded program under MATLAB (MathWorks, Inc., Natick, MA, USA) environment. Gait symmetry of all parameters were calculated by Symmetry Index (SI) and Gait Deviation Index (GDI) (21, 22).

Symmetry Index was calculated according to the formula (23):


$$\begin{array}{l} SI=\frac{2\left(X_{R}-X_{L}\right)}{X_{R}+X_{L}}\times 100\% \end{array}$$

X_R_: right leg parameter

X_L_: left leg parameter

* Signs before the SI value indicate the direction of asymmetry: negative means to the left, positive to the right. SI = 0 indicates full symmetry.

Changes in the gait pattern of each subject were categorized into one of the three following groups according to the change of GDI between visits (23, 24): improvement (ΔGDI ≥ 5), no change (−5 < ΔGDI < 5), or deterioration (ΔGDI ≤−5).

## Results

### Spatiotemporal Parameters

After receiving RAT, we did not find statistically significant changes in spatiotemporal parameters neither right after therapy (t2) nor 6 weeks after therapy (t3). The same analysis was done in functional subgroups and no statistically significant changes were found either (Table 1).

**Table 1:** Changes in spatiotemporal parameters between visits.

**Parameter**	**NAS** **+** **AS (*****n*** **=** **26)**				**NAS (*****n*** **=** **17)**				**AS (*****n*** **=** **9)**			
	**Mean** **(SD)** **t1**	**Mean** **(SD)** **t2**	**Mean** **(SD)** **t3**	***p***	**Mean** **(SD)** **t1**	**Mean** **(SD)** **t2**	**Mean** **(SD)** **t3**	***p***	**Mean** **(SD)** **t1**	**Mean** **(SD)** **t2**	**Mean** **(SD)** **t3**	***p***
Cadence (step/min)	84.32 (25.87)	85.36 (24.0)	85.80 (23.23)	0.41	94.71 (16.73)	95.06 (12.41)	97.80 (9.05)	0.79	64.70 (29.49)	67.04 (30.23)	63.13 (25.28)	0.74
Double support (s)	0.73 (0.99)	0.66 (0.79)	0.65 (0.77)	0.41	0.43 (0.29)	0.40 (0.19)	0.34 (0.11)	0.26	1.30 (1.53)	1.16 (1.21)	1.23 (1.11)	0.46
Foot off (%)	67.29 (8.85)	67.01 (9.38)	66.87 (8.32)	0.15	65.02 (5.14)	64.91 (4.79)	63.46 (3.34)	0.08	71.57 (12.66)	70.97 (14.22)	73.32 (11.06)	0.60
Opposite foot contact (%)	49.94 (0.76)	50.22 (0.88)	50.11 (0.65)	0.40	49.85 (0.73)	50.02 (0.69)	50.01 (0.63)	0.69	50.11 (0.84)	50.58 (1.11)	50.31 (0.67)	0.89
Opposite foot off (%)	17.38 (9.24)	17.30 (9.57)	17.01 (8.22)	0.45	15.10 (5.36)	14.97 (4.39)	13.66 (3.40)	0.33	21.67 (13.32)	21.69 (14.64)	23.35 (10.91)	0.72
Single support (s)	0.48 (0.14)	0.47 (0.12)	0.47 (0.08)	0.96	0.45 (0.06)	0.45 (0.05)	0.45 (0.04)	0.90	0.54 (0.23)	0.52 (0.19)	0.51 (0.11)	0.97
Step length (m)	0.45 (0.14)	0.44 (0.15)	0.45 (0.15)	0.63	0.47 (0.15)	0.48 (0.13)	0.49 (0.13)	0.31	0.41 (0.13)	0.37 (0.17)	0.36 (0.15)	0.61
Step time (s)	0.85 (0.49)	0.80 (0.37)	0.79 (0.38)	0.97	0.67 (0.18)	0.65 (0.09)	0.62 (0.06)	0.90	1.19 (0.69)	1.08 (0.51)	1.12 (0.51)	0.56
Step width (m)	0.19 (0.07)	0.18 (0.07)	0.19 (0.07)	0.82	0.20 (0.07)	0.20 (0.08)	0.20 (0.08)	0.94	0.15 (0.04)	0.15 (0.03)	0.16 (0.05)	0.69
Stride length (m)	0.87 (0.31)	0.88 (0.30)	0.89 (0.31)	0.56	0.95 (0.29)	0.95 (0.26)	0.99 (0.27)	0.33	0.73 (0.33)	0.73 (0.33)	0.72 (0.32)	0.46
Stride time (s)	1.69 (1.0)	1.60 (0.76)	1.59 (0.77)	0.96	1.33 (0.36)	1.29 (0.19)	1.24 (0.12)	0.65	2.39 (1.42)	2.20 (1.06)	2.25 (1.04)	0.64
Walking speed (m/s)	0.65 (0.34)	0.66 (0.32)	0.68 (0.33)	0.65	0.77 (0.31)	0.77 (0.27)	0.82 (0.26)	0.63	0.44 (0.29)	0.47 (0.33)	0.43 (0.29)	0.46

*t1, before therapy; t2, right after therapy; t3, 6 weeks after therapy*.

### Gait Symmetry

**Figure 1:**
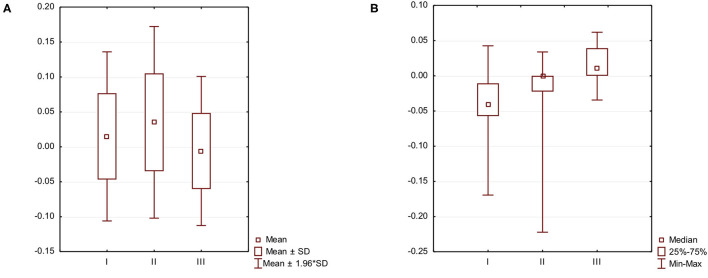
Gait symmetry: **(A)** spatiotemporal parameter symmetry for Group NAS + AS; **(B)** walking speed symmetry for Group AS.

Analysis of SI for spatiotemporal parameters showed significant changes in double support symmetry while considering all participants as one group, i.e., NAS + AS (t2 = 0.04, t3 = −0.01; *p* = 0.03; Figure 1A), and walking speed symmetry for patient walking with aids, i.e., AS (t1 = −0.04, t3 = 0.02; *p* = 0.02; Figure 1B) (Table 2).

**Table 2:** Changes in gait symmetry between tests.

**Parameter**	**NAS** **+** **AS (*****n*** **=** **26)**				**NAS (*****n*** **=** **17)**				**AS (*****n*** **=** **9)**			
	**Mean** **(SD)** **t1**	**Mean** **(SD)** **t2**	**Mean** **(SD)** **t3**	***p***	**Mean** **(SD)** **t1**	**Mean** **(SD)** **t2**	**Mean** **(SD)** **t3**	***p***	**Mean** **(SD)** **t1**	**Mean** **(SD)** **t2**	**Mean** **(SD)** **t3**	***p***
Cadence (step/min)	−0.01 (0.02)	−0.01 (0.04)	0.00 (0.03)	0.81	−0.01 (0.02)	−0.01 (0.03)	0.00 (0.02)	0.69	−0.01 (0.03)	−0.01 (0.05)	0.00 (0.03)	0.26
Double support (s)	0.01 (0.06)	0.04 (0.07)	−0.01 (0.05)	0.03^*^	0.00 (0.05)	0.04 (0.08)	0.00 (0.06)	0.05	0.04 (0.08)	0.03 (0.06)	−0.02 (0.04)	0.16
Foot off (%)	−0.02 (0.18)	0.00 (0.17)	0.00 (0.12)	0.54	−0.03 (0.09)	−0.02 (0.08)	−0.02 (0.07)	0.94	0.01 (0.28)	0.03 (0.27)	0.05 (0.17)	0.24
Opposite foot contact (%)	−0.02 (0.22)	−0.02 (0.19)	−0.03 (0.17)	0.45	−0.06 (0.15)	−0.05 (0.15)	−0.06 (0.10)	0.66	0.06 (0.30)	0.04 (0.27)	0.03 (0.25)	0.61
Opposite foot off (%)	−0.02 (0.33)	−0.03 (0.31)	−0.13 (0.31)	0.81	−0.07 (0.27)	−0.04 (0.13)	−0.13 (0.21)	0.33	0.08 (0.42)	−0.02 (0.35)	−0.13 (0.47)	0.27
Single support (s)	−0.04 (0.29)	−0.02 (0.26)	0.01 (0.23)	0.36	−0.07 (0.18)	−0.05 (0.31)	−0.03 (0.16)	0.33	0.02 (0.43)	0.04 (0.42)	0.10 (0.32)	0.64
Step length (m)	−0.06 (0.31)	−0.07 (0.48)	0.07 (0.37)	0.23	−0.06 (0.30)	−0.05 (0.13)	−0.02 (0.23)	0.90	−0.06 (0.37)	−0.12 (0.75)	0.24 (0.52)	0.14
Step time (s)	0.02 (0.22)	0.03 (0.19)	0.03 (0.16)	0.34	0.07 (0.16)	0.06 (0.29)	0.06 (0.09)	0.91	−0.06 (0.30)	−0.03 (0.27)	−0.02 (0.25)	0.68
Step width (m)	0.02 (0.08)	0.01 (0.10)	−0.02 (0.13)	0.47	0.02 (0.09)	0.00 (0.14)	−0.03 (0.15)	0.36	0.03 (0.06)	0.03 (0.07)	0.01 (0.09)	0.70
Stride length (m)	0.00 (0.05)	0.01 (0.04)	0.01 (0.03)	0.16	0.01 (0.03)	0.01 (0.12)	0.00 (0.02)	0.32	−0.02 (0.06)	0.01 (0.05)	0.02 (0.04)	0.10
Stride time (s)	0.01 (0.02)	0.01 (0.04)	0.00 (0.03)	0.76	0.01 (0.02)	0.01 (0.03)	0.00 (0.02)	0.66	0.02 (0.03)	0.01 (0.05)	0.00 (0.03)	0.80
Walking speed (m/s)	−0.01 (0.05)	−0.01 (0.05)	0.01 (0.03)	0.47	0.00 (0.03)	0.00 (0.03)	0.00 (0.02)	0.83	−0.04 (0.06)	−0.03 (0.07)	0.02 (0.03)	0.02^*^

*t1, before therapy; t2, right after therapy; t3, 6 weeks after therapy*;

* *Statistically significant*.

### Gait Deviation Index

Taking all the 26 participants as one group, the GDI at baseline (t1) was: mean (SD) = 73.36 (11.28), min = 55.19, max = 100.52, median = 71.56. We did not find any significant change in GDI neither right after therapy (t2: mean [SD] = 72.62 [12.20], min = 53.45, max = 102.39, median = 72.17) nor 6 weeks after the last session (t3: mean [SD] = 74.02 [13.29], min = 56.63, max = 102.71, median = 71.11) (*p* = 0.45).

While focused on the change of GDI (improvement, no change, or deterioration), the analysis showed that changes right after therapy (t2) and after 6 weeks (t3) were not statistically significant (Table 3). The same analysis was performed based on functional subgroups. No significant change was found in either Group NAS (*p* = 0.80) or Group AS (*p* = 0.92).

**Table 3:** Number of participants who had changes in GDI.

**Category**	**t2 vs. t1** ***n* = 26**	**t3 vs. t1** ***n* = 26**
Improvement	4 (15.4%)	7 (26.9%)
Deterioration	6 (23.1%)	6 (23.1%)
No changes	16 (61.5%)	13 (50%)

Analysis of GDI symmetry between left and right sides did not show statistically significant changes between visits neither for the whole study group (NAS + AS, *p* = 0.31), nor for subgroups (NAS, *p* = 0.40; AS, *p* = 0.36).

Comparing differences between the two groups, the statistical power was in the range 32.3–74.2%. Comparing the differences in time, the statistical power was in the range of 91.0–92.0%.

## Discussion

The primary aim of the study was to assess the effects of RAT on selected objective gait parameters in hypertonic children with CP. We did not find any significant effect of RAT on majority of gait parameters except for the improvement in walking speed symmetry (Group AS: t1 vs. t3, *p* = 0.02) and improvement in double support symmetry (Group AS: t2 vs. t3, *p* = 0.03). At t1, there was a slight asymmetry to the right side of the body (SI = 0.01). Right after the therapy (t2), the asymmetry shifted more toward the right side of the body (SI = 0.04). At t3, the asymmetry shifted to the opposite direction to the left (SI = −0.01). The secondary aim of the study was to determine whether changes in selected objective gait parameters are different among children in different ambulatory levels. We did not find differences in changes in selected objective gait parameters between children walking with or without aids.

### Therapeutic Effects of RAT

A significant benefit from the RAT was only noticed in two parameters of walking symmetry. It is not clear if this limited, although statistically significant improvement in symmetry of walking has any impact on the clinical or functional condition of a patient. The possible explanation of these improvements is our training protocols. Except pure gait training, the therapy was focused on load distribution between body sides (gamma dynamographic platforms) and balancing of center of pressure in all directions (alfa stabilometric platform). Patients were trained with virtual reality games to gain better control of the loading of body weight on the left and right foot. Moreover, the exoskeleton force subjects to take more regular steps. It can explain the prominent changes in some parameters of gait symmetry. Surprisingly, we did not notice any improvement in spatiotemporal parameters and pattern of walking.

It is worth emphasizing that despite the fact that changes in GDI were not statistically significant, we still observed improvements in some patients (t2 = 15.4%; t3 = 26.9%). Specific analysis on patients whose gait pattern improved or deteriorated could provide the answer who will possibly benefit from this kind of therapy program the most.

In addition, outcome measures used in this study were highly variable, which was confirmed by non-parametric distribution. The variability was conspicuous especially in Group AS. The explanation of such highly scattering results can be due to individuals with all levels of the ambulation abilities (i.e., GMFCS levels I to IV) were included.

### Inconsistency to Previous Studies

Our findings are in contrary to the majority of previous reports. Previous studies reported significant benefits of RAT for the patient with neurological deficits, including improvement of gait parameters, balance and functional status (changes of gross motor function measure [GMFM] total score and dimensions D & E scores). Important changes were shown in self-paced gait velocity of walking, step length, cadence, muscles activity, and kinematic data of the knee movement (25–30). In contrary, our data between three visits (t1, t2, and t3) showed that neither right after therapy (t2) nor 6 weeks after the last session (t3), changes in the kinematics of gait (GDI), symmetry (assessed for all parameters) and spatiotemporal parameters did not occur. Bayón et al. showed that better benefits were achieved by more affected individuals than those on higher functional level (29). According to our results, there were no changes in the most outcome measures at t2 or t3 among children in different ambulatory levels, except speed symmetry, which improves only in the more affected group, i.e., the Group AS. Mean value of spatiotemporal parameters, SI or GDI did not change neither in NAS nor in AS which suggested that functional status of subjects and effects of RAT might not be directly related. We believe these disagreements in findings could be explained:

First, the sample size of research group improvement in spatiotemporal parameters was shown in studies based on a relatively small number of participants (26–31). The impact of ambulation level on therapy benefits was shown in group of 4 merely subjects (29). There was only one study with a similar number of patients to our study, where therapy was based on locomotor treadmill. However, no significant changes in spatiotemporal parameters were found in this study either (12). Our study groups consist of 26 subjects with all levels of ambulatory function (GMFCS levels I to IV) which may reflect the therapeutic effect to individuals affected by different severity of CP.

Second, the training and examination protocols are different from study to study. Some researchers showed data collected during the therapy session (improvement in crouch gait, electromyographic activity) and proved that the equipment can change the pattern of gait, but did not show its effectiveness for daily walking ability (12, 31–36). The effects of RAT analysis vary from 1 to 20 session which were conducted from five visits over 12 weeks to five times per week. The therapy in our study lasted for 10 weeks and each patient received 30 training sessions, five sessions per week. Moreover, we decided to combine gait and balance training as strongly indissoluble activities. The short-term effect of the RAT was evaluated twice: right after therapy was completed and 6 weeks later.

Last, the equipment varies from study to study. The effectiveness of different kinds of an exoskeleton, CP walkers, treadmills, and virtual reality training sometimes combined with conventional therapy, were used. In our study, patients received combined therapy of exoskeleton, treadmill, platforms, and virtual reality video game, all within one session. Our intention was to assess pure RAT. In theory, this kind of intervention is supposed to understand needs and requirements of the subject easier than a conventional one. Also, it is easier to adjust the training program to demands of an individual.

### Limitations

One major limitation of this study is the data analysis method used. We have used an average of three trials. For those patients whose gait pattern is very consistent, it is a reasonable method. However, in our research group, we had patients at GMFCS level IV whose stride-to-stride variability is commonly high. Bulea et al. suggested using the variance ratio for kinematic data processing to evaluate the repeatability of results in a more reasonable way in these cases (36).

No matched control group was used in the present study due to the clinical restriction. A full-scale case-control study will help to clarify unanswered questions.

Results of this study do not provide strong evidence to support that RAT can be highly beneficial for individuals with spastic CP in improving gait functions. However, the length of follow-up was only after one training protocol, and only part of the gait parameters was investigated. We did not take the endurance of walking or general gross motor function into consideration. Future research projects on the effect of therapy intensity with similar protocols and other outcome measures are required to clarify the most suitable candidates and the optimal therapy dose for RAT. Moreover, it is also very important to evaluate electromyography, to assess the effect on possible neural structure reorganization.

## Conclusion

Our data suggest that RAT based on our protocol does not change spatiotemporal parameters and kinematics of walking except for limited improvement in some aspects of gait symmetry. Moreover, we did not find differences in changes in selected objective gait parameters among children in different ambulatory levels.

## Data Availability Statement

The data that support the findings of this study are available from the corresponding author upon reasonable request. The data are not publicly available due to privacy or ethical restrictions.

## Ethics Statement

The studies involving human participants were reviewed and approved by Ethical Committee—Poznań University of Medical Sciences, Poland. Written informed consent to participate in this study was provided by the participants' legal guardian.

## Author Contributions

FM: contributes to the study concept and design, data collection, data analysis, manuscript drafting, and final manuscript approval. SB and AK: contributes to the data collection, data analysis, and final manuscript approval. MJ: contributes to the study design, data analysis, and final manuscript approval. All the authors have read and agreed to the published version of the manuscript.

## Funding

This study was supported by Polish/National Center for Research and Development (NCBR; Grant # RPWP.01.02.00-30-0118/17).

## Conflict of Interest

The authors declare that the research was conducted in the absence of any commercial or financial relationships that could be construed as a potential conflict of interest.

## Publisher's Note

All claims expressed in this article are solely those of the authors and do not necessarily represent those of their affiliated organizations, or those of the publisher, the editors and the reviewers. Any product that may be evaluated in this article, or claim that may be made by its manufacturer, is not guaranteed or endorsed by the publisher.
